# RNA-seq reveals post-transcriptional regulation of *Drosophila* insulin-like peptide *dilp8* and the neuropeptide-like precursor *Nplp2* by the exoribonuclease Pacman/XRN1

**DOI:** 10.1093/nar/gkv1336

**Published:** 2015-12-09

**Authors:** Christopher I. Jones, Amy L. Pashler, Benjamin P. Towler, Sophie R. Robinson, Sarah F. Newbury

**Affiliations:** Medical Research Building, Brighton and Sussex Medical School, University of Sussex, Falmer, Brighton BN1 9PS, UK

## Abstract

Ribonucleases are critically important in many cellular and developmental processes and defects in their expression are associated with human disease. Pacman/XRN1 is a highly conserved cytoplasmic exoribonuclease which degrades RNAs in a 5′-3′ direction. In *Drosophila*, null mutations in *pacman* result in small imaginal discs, a delay in onset of pupariation and lethality during the early pupal stage. In this paper, we have used RNA-seq in a genome-wide search for mRNAs misregulated in *pacman* null wing imaginal discs. Only 4.2% of genes are misregulated ±>2-fold in *pacman* null mutants compared to controls, in line with previous work showing that Pacman has specificity for particular mRNAs. Further analysis of the most upregulated mRNAs showed that Pacman post-transcriptionally regulates the expression of the secreted insulin-like peptide Dilp8. Dilp8 is related to human IGF-1, and has been shown to coordinate tissue growth with developmental timing in *Drosophila*. The increased expression of Dilp8 is consistent with the developmental delay seen in *pacman* null mutants. Our analysis, together with our previous results, show that the normal role of this exoribonuclease in imaginal discs is to suppress the expression of transcripts that are crucial in apoptosis and growth control during normal development.

## INTRODUCTION

Control of gene expression at the post-transcriptional level and control of mRNA stability are well recognized as important cellular processes in eukaryotic organisms. The effect of controlled RNA turnover on gene expression can be extremely significant: for example, some studies have shown that 40–50% of changes in gene expression occur at the level of RNA stability (1). In multi-cellular organisms, it is increasingly evident that degradation of specific mRNAs is critical for the regulation of many cellular processes, including early development, infection and inflammation, apoptosis and ageing (2–4). Disruption of gene expression at this level can lead to disease and developmental phenotypes, for example as occurs in mice deficient for the RNA binding protein AUF1, where the impaired ability to degrade RNAs such as TNFα and Interleukin-1β results in symptoms of septic shock in response to endotoxins (5,6). For this reason, it is vital to understand the role played by proteins involved in these processes, such as RNA degradation enzymes.

A number of cytoplasmic degradation enzymes are able to degrade RNAs in the 3′-5′ direction, but only one, XRN1 (Pacman in *Drosophila*), is able to degrade in the 5′-3′ direction (3,4). This lack of redundancy, and its important roles in RNA interference, nonsense-mediated decay and miRNA function (7,8) place XRN1 in a pivotal position in the RNA degradation pathway. Modulation of XRN1 availability and function has the potential to have widespread effects on cellular gene expression programmes and is already known to be critical in response to environmental factors and in disease. For example, XRN1 homologues are known to be required in developmental pathways, such as epithelial sheet sealing in *Caenorhabditis elegans* (9), are important in the cellular response to certain RNA viruses (10) and have been implicated in the development of osteosarcoma (11). The high level of conservation of XRN1 across eukaryotes highlights its importance, and offers the opportunity to elucidate the role of XRN1 and post-transcriptional control of gene expression in organisms such as *Drosophila melanogaster* while maintaining direct relevance to higher eukaryotes.

Previous work on Pacman in *Drosophila* (12–16) has shown that it is required for the correct growth and differentiation of a number of tissues including the wing imaginal discs, which develop into the wing blade, hinge and dorsal thorax of the adult. Pacman mutations also restrict the growth of other imaginal discs, as well as causing defects in thorax closure and reducing male and female fertility (13,15–17). Our previous results show that the hypomorphic *pcm^5^* mutation and the null *pcm^14^* mutation result in wing imaginal discs that are respectively 81.7% and 45.0% the size of control discs (14,15). The reduction in size of *pcm^14^* imaginal discs is accompanied by a post-transcriptional increase in the pro-apoptotic mRNAs *reaper* (*rpr*) and *hid* resulting in a massive increase in apoptosis in the wing pouch of the wing disc. In addition, the *pcm^14^* mutation results in a significant delay at the L3 stage of larval development in an attempt to allow the imaginal discs time to reach an appropriate size before pupariation (15). The loss of coordination between larval body size and the growth of the imaginal discs suggests that Pacman plays a role in controlling the coupling between body size and the growth of internal tissues.

In this publication, we have, for the first time, used RNA-seq to identify the biological pathways within the wing imaginal discs that are sensitive to the loss of Pacman. Using this genome-wide approach to compare two *pacman* null mutants with their respective wild-type controls we have identified the RNAs that are significantly differentially expressed in the mutant wing imaginal discs. Many genes involved in imaginal disc development and control of transcription were downregulated in the *pacman* null mutants, whereas genes involved in apoptosis and the immune response were upregulated. Interestingly, the *dilp8* gene, which is associated with developmental delay in response to retarded imaginal disc growth, was one of the most upregulated genes, suggesting a link between control of mRNA decay and tissue homeostasis.

## MATERIALS AND METHODS

### Fly stocks and crosses

Fly stocks were cultivated on standard media at 25°C in uncrowded conditions. All the stocks used were from the Bloomington Stock Center.

The *pcm^15^* allele was created by imprecise P-element excision of *P{EP}pcm^G1726^* from stock 33263 (w* P{EP}pcm^G1726^). The wild-type control (*pcm^WT2^*) used with *pcm^15^* was a neutral excision of *P{EP}pcm^G1726^* created at the same time. Creation of *pcm^14^* and its associated wild-type control (*pcm^WT1^*) have been described previously (14,15).

### RNA-seq sample preparation and analysis

Wing imaginal discs were dissected from non-fluorescent wandering L3 larvae from the wild-type and *pacman* null stocks balanced over *FM7i, P{ActGFP}JMR3*. (e.g. *w^1118^ pcm^15^*/*FM7i, P{ActGFP}JMR3*). A total of 60 wing discs were extracted per sample for *pcm^WT1^* and *pcm^WT2^* and 120 per sample for *pcm^14^* and *pcm^15^* to give >3 μg RNA per sample upon extraction with a miRNeasy Micro kit (Qiagen, cat. no. 217084) and DNase treatment with the optional RNase-Free DNase kit (Qiagen, cat. no. 79254). RNA integrity was assessed as high based on clear ribosomal peaks on a Bioanalyzer 2100 (Agilent). A total of 3 μg of RNA per sample was sent to Oxford Gene Technology for conversion to cDNA using oligo-dT primers and sequencing on a HiSeq2000 lane using TruSeq v3 chemistry (Illumina), generating 10–17 million reads per sample. Initial quality control of samples was performed using FastQC v0.11.2 (http://www.bioinformatics.babraham.ac.uk/projects/fastqc/), followed by adapter removal using Scythe v0.993b (https://github.com/vsbuffalo/scythe) and quality trimming using Sickle v1.29 (https://github.com/najoshi/sickle). Reads were aligned to chromosomes X, Y, 2, 3 and 4 of the FlyBase *D*.*melanogaster* genome (r6.03 (18)) using TopHat v2.0.12 (19) and Bowtie v2.2.3 (20). FPKM values and differential expression comparisons were performed using Cufflinks (21). Alignment results are summarized in Supplementary Figure S1, alongside a summary of non-default parameters used for the alignment and quantification. Further analysis was performed in R v3.1.2 and GraphPad Prism 6. RNA-seq data has been deposited in the ArrayExpress repository, accession no. E-MTAB-3789.

### qRT-PCR

cDNA reactions were performed with 100 ng of total RNA in duplicate using a High Capacity cDNA Reverse Transcription Kit (Life Technologies, cat. no. 4368814) and primers appropriate for the RNA to be tested (oligo-dT primers for mRNAs and random primers for pre-mRNAs and *Adh^fn6^* fragments).

All qRT-PCRs were performed in duplicate on each cDNA preparation (four technical replicates in total per sample/assay combination) using pre-designed TaqMan assays for mRNAs and custom assays for pre-mRNAs with TaqMan Universal PCR Master Mix, No AmpErase UNG (Life Technologies, cat. no. 4324018). Custom pre-mRNA assays are shown in Supplemental Figure S2.

### Immunocytochemistry

Immunocytochemistry was performed as described in (22). Primary antibodies used were rabbit anti-Cleaved Caspase-3 (Asp175) (Cell Signalling, cat no. 9661) at 1:400 dilution and rat anti-Dilp8 at 1:400 dilution (22). Secondary antibodies used were Cy-3-conjugated monoclonal goat anti-rabbit IgG (Jackson ImmunoResearch, cat. no. 711–165–152) at 1:400 dilution and anti-rat IgG Dylight 488 (Abcam, cat. no. ab96887) at 1:100 dilution. Images were taken with a Leica SP8 confocal microscope. Image stacks and 3D projections were processed in Fiji (http://fiji.sc/Fiji).

### SUnSET labelling

Surface sensing of translation (SUnSET) was performed on wild-type (*pcm^WT1^*) and *pacman* null (*pcm^14^*) wing imaginal discs. Wing discs were dissected in batches of 30 and incubated in Shields and Sang M3 insect medium (Sigma-Aldrich, cat. no S8398) containing 2μg/ml puromycin (Sigma-Aldrich, cat. no. P8833) for 2 h at 25°C. Western blotting was performed to determine the levels of puromycin incorporation with Tubulin as a loading control. Mouse anti-Tubulin primary antibody (Sigma-Aldrich, cat. no. T9026) was used at 1:2000 dilution. Mouse anti-Puromycin (clone 12D10) primary antibody (Merck Millipore, cat. no. MABE343) was used at 1:1000 dilution. Anti-mouse IRDye 800CW secondary antibody (LI-COR Biosciences, cat. no 926–32210) was used at 1:20 000 dilution to detect both primary antibodies. Each sample was run in parallel on two gels/membranes so the Tubulin band could be distinguished from puromycin containing peptides. Quantification of Tubulin (∼50 kDa) and puromycin peptides (from smallest size visible to 245 kDa) was achieved using LI-COR Biosciences Image Studio software.

## RESULTS

### Generation of a new *pacman* null allele

**Figure 1.**
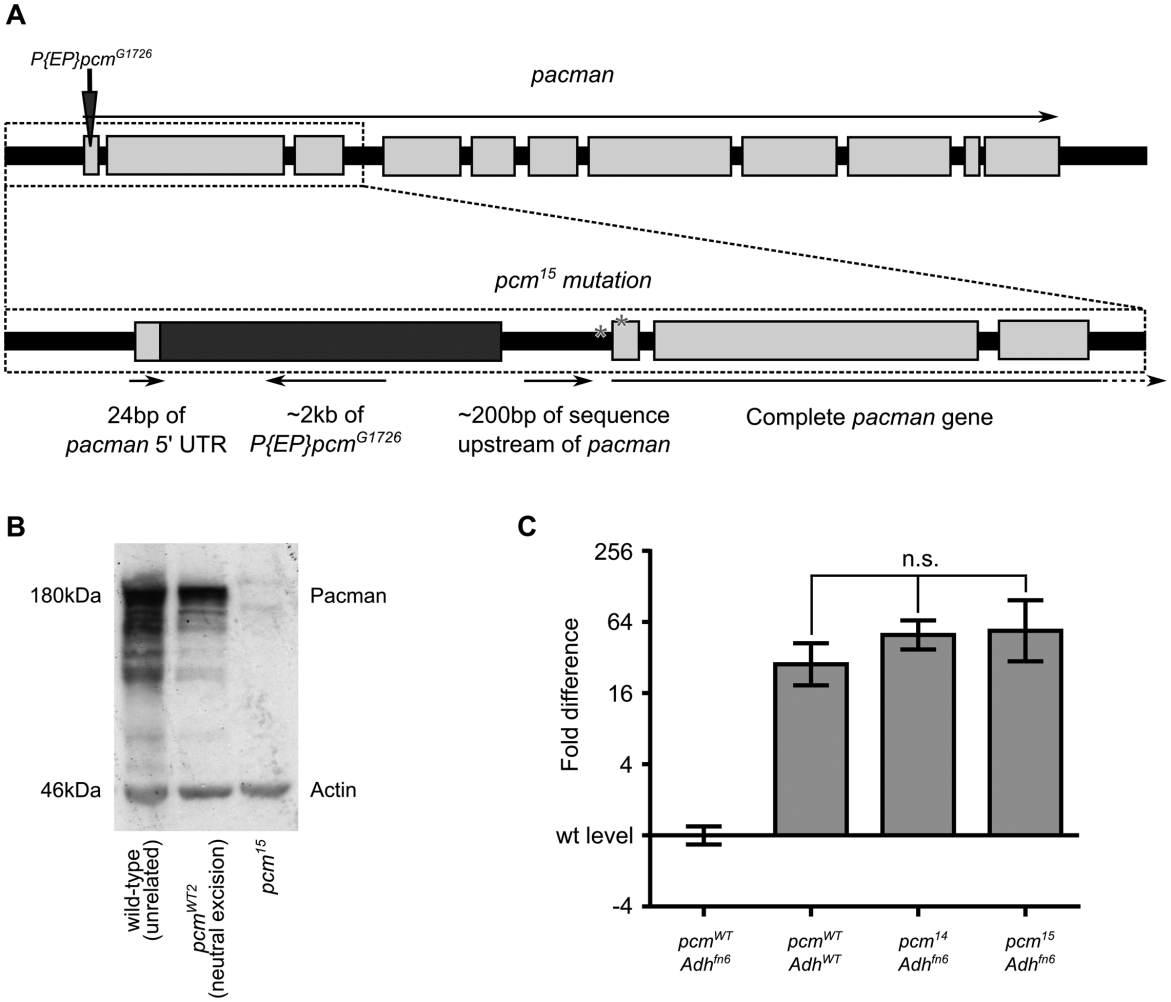
Characterization of the *pcm^15^* allele. (**A**) The *pcm^15^* allele was created by excision of the P-element *P{EP}pcm^G1726^* located 24 bp into the 5′ UTR of *pacman*. Excision of *P{EP}pcm^G1726^* was incomplete and also resulted in duplication of some of the 5′ UTR of *pacman* and upstream genomic sequence. Following this, a complete copy of the *pacman* gene was present with two single base changes, one 54 bp upstream of the gene (T ≥ C) and one 17 bp within the 5′ UTR (C ≥ T) (asterisks). (**B**) A strong Pacman band is present at around 180 kDa in *pcm^WT2^*, a line from which *P{EP}pcm^G1726^* has been neutrally excised, and in an unrelated wild-type line. No detectable Pacman protein is expressed from the *pcm^15^* allele. (**C**) *Adh^fn6^* is an allele of *Alcohol dehydrogenase* that undergoes NMD and is cleaved into 5′ and 3′ fragments. Pacman degrades the 3′ fragment. In the null *pcm^14^* allele, the 3′ fragment of *Adh^fn6^* is present at the same level as wild-type *Adh* as no functioning Pacman protein is present. The same occurs for *pcm^15^*, showing that *pcm^15^* is also a null *pacman* allele. *n* = 12 for *pcm^WT^ Adh^fn6^* and *pcm^WT^ Adh^fn6^*,*n* = 11 for *pcm^14^ Adh^fn6^*, *n* = 9 for *pcm^15^ Adh^fn6^*. One-way ANOVA with Tukey's post-test was used to compare groups. *P* < 0.0001 for comparisons between *pcm^WT^ Adh^fn6^* and any other group, *P* > 0.05 for any comparisons between *pcm^WT^ Adh^WT^, pcm^14^ Adh^fn6^* and *pcm^15^ Adh^fn6^*. Error bars show 95% confidence intervals.

In previous work, we utilized the null *pacman* allele *pcm^14^*, which was created by excision of a P-element located downstream of the *pacman* locus, within the intron of *CR42360*, a non-coding RNA. The deletion extending into the *pacman* locus in *pcm^14^* also removes the 5′ of *CR42360*. Comparison with *pcm^13^*, a viable *pacman* allele in which *CR42360* was completely deleted, indicated that loss of *CR42360* had no discernible phenotypic effect (15). Additionally, *CR42360* is not widely expressed and is not expressed in wing imaginal discs (23), the tissue on which previous work has focused. However, to address any potential concerns about this additional factor in the *pcm^14^* mutation, we created a new null *pacman* allele, *pcm^15^*, by excision of the P-element *P{EP}pcm^G1726^*, located 24 bp within the 5′ UTR of *pacman*. Characterization of *pcm^15^* revealed a complicated partial excision of *P{EP}pcm^G1726^* and duplication of genomic sequence (Figure 1A) rather than a simple deletion as expected. The effect of the mutation was consistent with *pcm^15^* being a null allele however, as no Pacman protein could be detected in homozygous *pcm^15^* larvae, despite a complete copy of the *pacman* gene being present (Figure 1B).

To confirm *pcm^15^* as a null mutation, we made use of an assay we have previously employed to assess the *in vivo* activity of the Pacman protein (15). This assay makes use of an allele of Alcohol dehydrogenase, *Adh^fn6^*, which is known to be subject to nonsense-mediated decay (24,25). The extent of 5′-3′ degradation of the cleaved 3′ fragment as measured by qRT-PCR, provides a quantitative measurement of the 5′-3′ activity of Pacman *in vivo*. As for *pcm^14^*, the *pcm^15^* mutant showed no ability to degrade this 3′ fragment of *Adh^fn6^* (Figure 1C), demonstrating that it is a null allele. This is consistent with the phenotype of the *pcm^15^* homozygotes, which is indistinguishable from that of *pcm^14^* homozygotes (15) in that larvae have small wing imaginal discs due to increased apoptosis and die as pupae after an extended period as L3 larvae. A stock from which *P{EP}pcm^G1726^* was neutrally excised (without affecting *pacman* expression) was also retained for use as a wild-type control from *pcm^15^*, referred to as *pcm^WT2^*.

### RNA-sequencing shows that Pacman affects expression of a small number of genes

**Figure 2.**
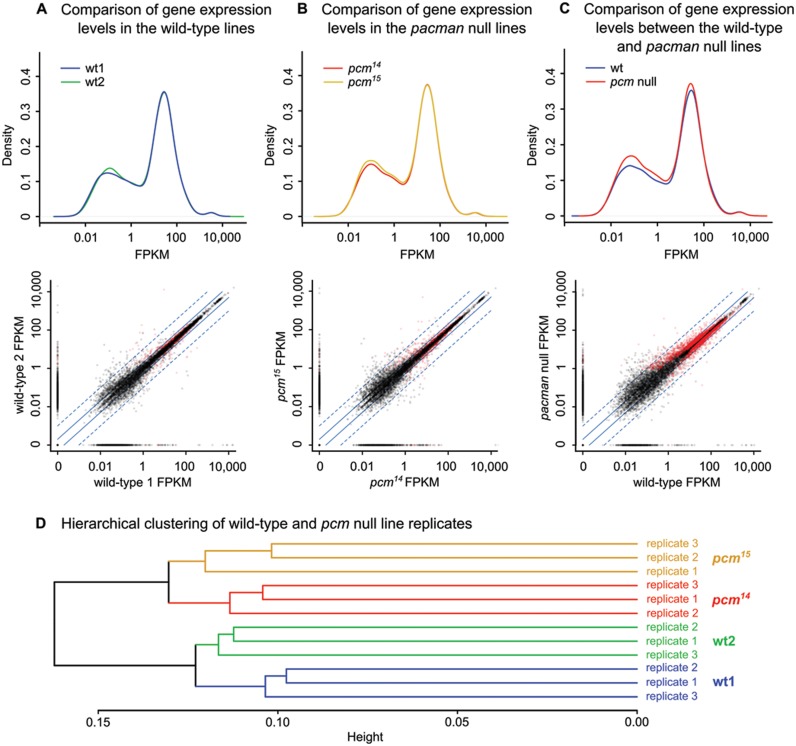
Comparisons of RNA-seq gene expression profiles. (**A**) Comparison of the overall gene expression profiles of the wild-type lines shows very little difference between *pcm^WT1^* and *pcm^WT2^*. The scatterplot below shows that only 258 genes vary significantly (1.6% of 16 244). (**B**) Comparison of the overall gene expression profiles of the *pacman* null lines shows very little difference between the *pcm^14^* and *pcm^15^*. The scatterplot below shows that only 386 genes vary significantly (2.4% of 16 244). (**C**) When wild-type samples and *pacman* null samples are grouped together, there are evident differences in the gene expression profiles of the wild-type and *pacman* null groups. More genes are detected at low levels (0.01 – 1 FPKM) and moderate levels (1 – 100 FPKM) in the *pacman* null group. The scatterplot below shows the extent of gene expression differences as 2498 genes (15.4% of 16 244) are significantly differentially regulated between groups. For Kernel density plots, ‘Density’ represents probability per log_10_(FPKM). For the scatterplots, red dots indicate significantly differentially regulated genes reported by Cufflinks, solid blue lines represent ±2-fold expression difference and dashed blue lines represent ±10-fold expression difference. *n* = 3 for each line (*pcm^WT1^*, *pcm^WT2^, pcm^14^* and *pcm^15^*). (**D**) Dendrogram showing close hierarchical clustering between the *pacman* wild-type lines and between the *pacman* null lines.

To identify the RNAs that are misregulated in *pacman* null mutants, and are likely to cause the above phenotypes, we used genome-wide RNA-sequencing (RNA-seq). This RNA-seq analysis was performed on *pcm^14^* and *pcm^15^*, and their respective wild-type controls (*pcm^WT1^* and *pcm^WT2^*) to globally analyse gene expression changes in the *pacman* null mutants. RNA was prepared in triplicate for each line from dissected wing imaginal discs, subjected to RNA-seq and analysed using Cufflinks (see ‘Materials and Methods’ section). To determine whether the gene expression levels were consistent between each of the two wild-type lines and also between the two *pacman* null lines, gene expression profiles were compared. Figure 2A shows the gene expression distributions of *pcm^WT1^* and *pcm^WT2^* and a scatterplot comparing individual gene levels in each line. A total of 258 genes (1.6% of 16 244) were reported as significantly differentially regulated by Cufflinks between the two wild-type lines (red dots), 156 of which were misregulated by ±>2-fold. Figure 2B shows the gene expression distributions of *pcm^14^* and *pcm^15^* and a scatterplot comparing individual gene levels in each line. A total of 386 (2.4% of 16 244) were reported as significantly differentially regulated by Cufflinks between the *pacman* null lines (red dots), 243 of which were misregulated by ±>2-fold. The gene expression profiles showed that the majority of apparent differences between *pcm^WT1^* and *pcm^WT2^* and between *pcm^14^* and *pcm^15^* were in the region of genes with FPKM <1, where technical variations are known to be greater. As this produced little evidence to suggest that the wild-type lines differed substantially from each other, or that the *pacman* null lines substantially differed from each other, the samples were grouped into a wild-type group and a *pacman* null group. Since each of these two groups comprised six biological replicate samples, this strategy increases the robustness of our results. Figure 2C shows the gene expression distributions of the combined wild-type and combined *pacman* null lines and a scatterplot comparing individual gene levels in each group. A total of 2498 (15.4% of 16 244) were reported as significantly differentially regulated by Cufflinks between the two groups (red dots), 676 of which were misregulated by ±>2-fold. Therefore only 4.2% of genes are misregulated ±>2-fold in *pacman* null mutants compared to controls, in line with our previous work showing that Pacman has specificity for particular mRNAs. Figure 2D shows hierarchical clustering of the *pcm^WT1^*, *pcm^WT2^*, *pcm^14^* and *pcm^15^* replicates, highlighting the similarity between *pcm^WT1^* and *pcm^WT2^* and between *pcm^14^* and *pcm^15^*.

**Figure 3.**
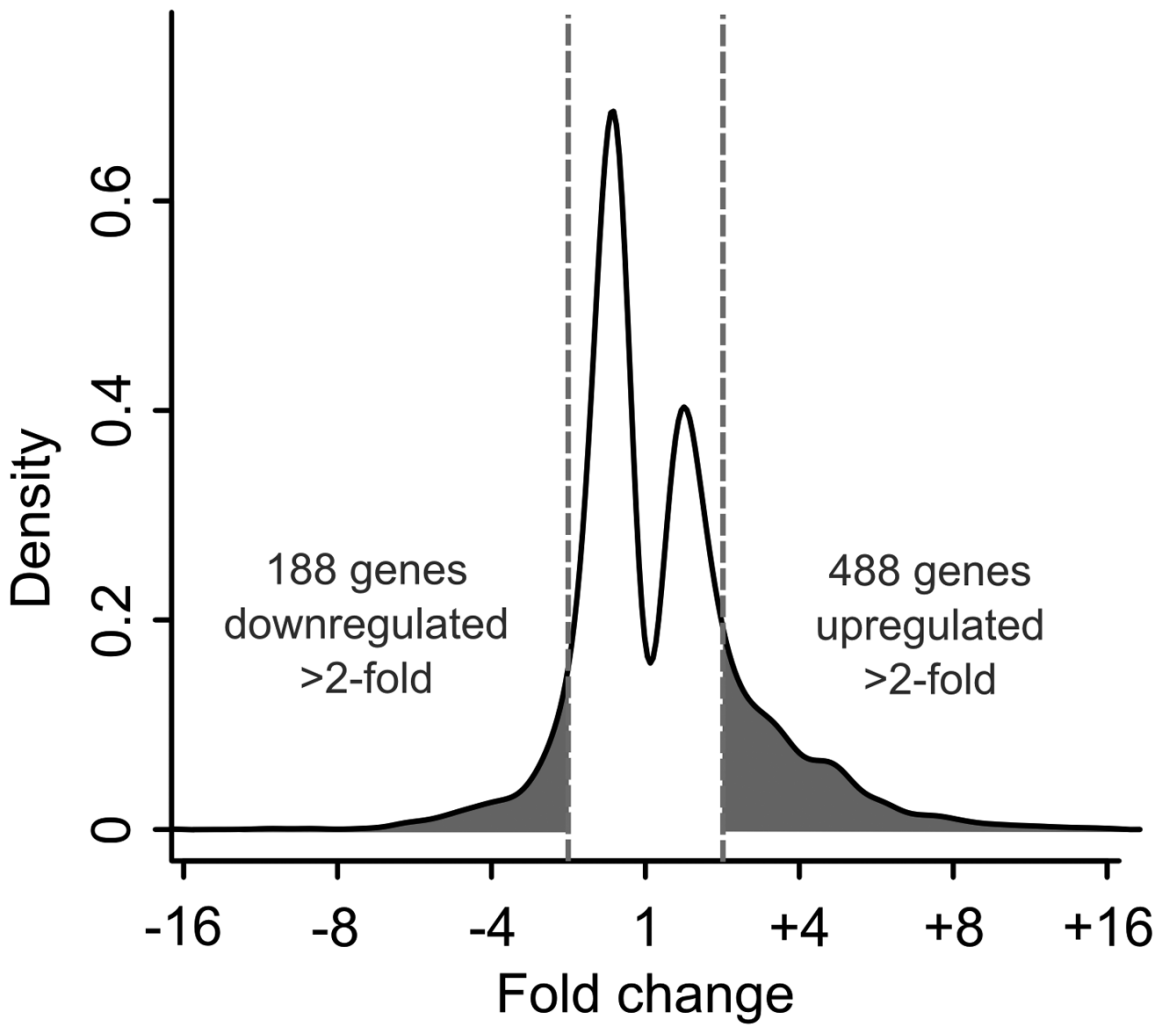
The absolute magnitudes of gene upregulation are greater than the magnitudes of gene downregulation in the *pacman* nulls. A total of 1207 genes are significantly upregulated and 1291 are significantly downregulated, but 488 genes are upregulated by >2-fold while 188 genes are downregulated >2-fold (shaded areas and dashed lines). ‘Density’ represents probability per log_2_(fold change).

Since Pacman is an exoribonuclease that degrades RNAs, it would be reasonable to expect that the candidate targets of Pacman will increase in expression in a *pacman* null mutant. RNAs that decrease in levels are more likely to be due to indirect effects. In the *pacman* null mutants, a similar number of genes are significantly upregulated (1207) as downregulated (1291), but the magnitudes of increases in expression tend to be greater than the magnitudes of decreases in expression. For example, 488 genes are upregulated >2-fold whereas 188 genes are downregulated >2-fold in the *pacman* null mutants compared to controls (Figure 3). This data is consistent with the role of Pacman as an exoribonuclease and confirms its normal role in suppressing the expression of a subset of RNAs.

### Gene Ontology analysis of differentially regulated genes

To identify the cellular pathways regulated by Pacman, we used the DAVID Gene Functional Classification tool (26) to classify genes that are significantly up- and downregulated ±>1.5-fold (Tables 1 and 2). Six categories are over-represented for the upregulated genes, including programmed cell death, in line with our previous work linking Pacman with apoptosis, and two immune response categories. For the downregulated genes, categories involving development and transcriptional regulation are strongly overrepresented, fitting with the developmental delay of *pcm^14^* mutants.

**Table 1.** Gene ontology analysis of genes upregulated by >1.5-fold in *pacman* null wing imaginal discs

Genes upregulated ≥1.5-fold (763)	
Term category	Enrichment score (-log10(*P*-value))
Immune response	2.20
Metabolic processes	1.65
Programmed cell death	1.57
Amino acid transport	1.42
Regulation of immune response	1.33
Ion transport	1.31

**Table 2.** Gene ontology analysis of genes downregulated by >1.5-fold in *pacman* null wing imaginal discs (top 10 categories)

Genes downregulated ≥1.5-fold (773)	
Term category	Enrichment score (-log10(*P*-value))
Imaginal disc development	17.35
Transcriptional regulation	14.21
Neuronal morphogenesis	11.60
Respiratory system development	11.60
Positive transcriptional regulation	7.17
Tissue morphogenesis	7.14
Eye development	6.87
Imaginal disc patterning	5.97
Hair cell development	5.41
Leg disc patterning	5.18

### Use of a novel ‘inconsistency index’ to assess the reliability of RNA-seq results

**Figure 4.**
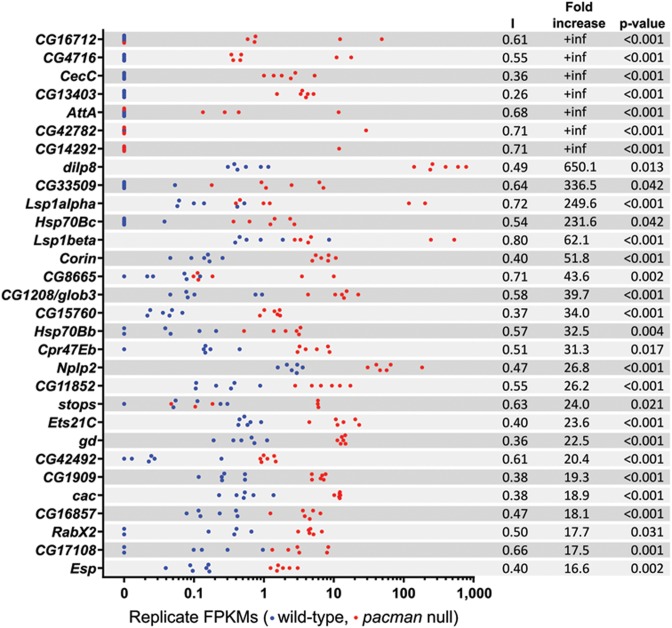
Strip plots of the top 30 significantly upregulated genes. Blue dots represent wild-type replicates and red dots represent *pacman* replicates. The inconsistency index (I) for each gene is shown alongside the fold increase and corrected *P*-value (*q*-value reported by Cufflinks).

**Figure 5.**
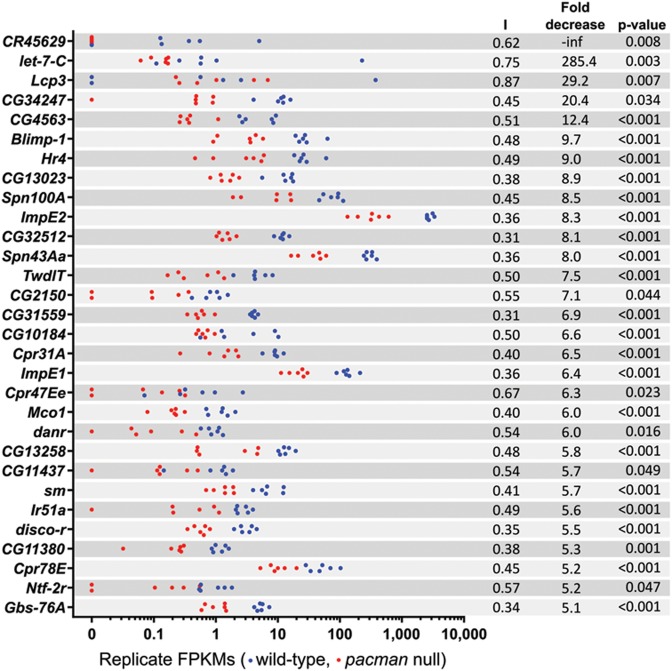
Strip plots of the top 30 significantly downregulated genes. Blue dots represent wild-type replicates and red dots represent *pacman* replicates. The inconsistency index (I) for each gene is shown alongside the fold decrease and corrected *P*-value (*q*-value reported by Cufflinks).

Our previous experiments using RNA-seq emphasized the importance of assessing the consistency of gene expression across biological replicates (27). This is particularly important when identifying gene expression changes to follow up which are biologically relevant. To assess the reliability of the gene expression changes reported by Cufflinks, we employed two methods to quantify the consistency of the FPKMs reported for the samples in each group. Strip plots were produced for the top 30 upregulated genes (Figure 4) and top 30 downregulated genes (Figure 5) to visually assess how well points for each group clustered together. The similarities in genes expression changes of the top 50 up- and downregulated genes in related samples are shown as a heat map in Supplemental Figure S3. Additionally, we developed an index to allow the variation of each gene to be compared without being biased by expression level. The index calculation is described in Supplemental Figure S4 and gives a value 0 < I ≤ 1 for each gene with higher values indicating greater inconsistency of expression. Genes with 0 < I ≤ 0.33 are very consistently expressed between replicates of the same type (e.g. *CG32512*, Figure 5), whereas genes with 0.33 < I ≤ 0.66 are consistently expressed (e.g. *dilp8*, Figure 4) and genes with 0.66 < I ≤ 1 are not consistently expressed (e.g. *Lsp1α*, Figure 4). By applying this ‘Inconsistency Index’ to the top 30 upregulated genes, we narrowed down the most likely Pacman targets to 16 for further validation by TaqMan qRT-PCR.

### qRT-PCR verification of the RNA-seq results shows that *dilp8* and *Nplp2* are highly upregulated in *pacman* null mutants

**Figure 6.**
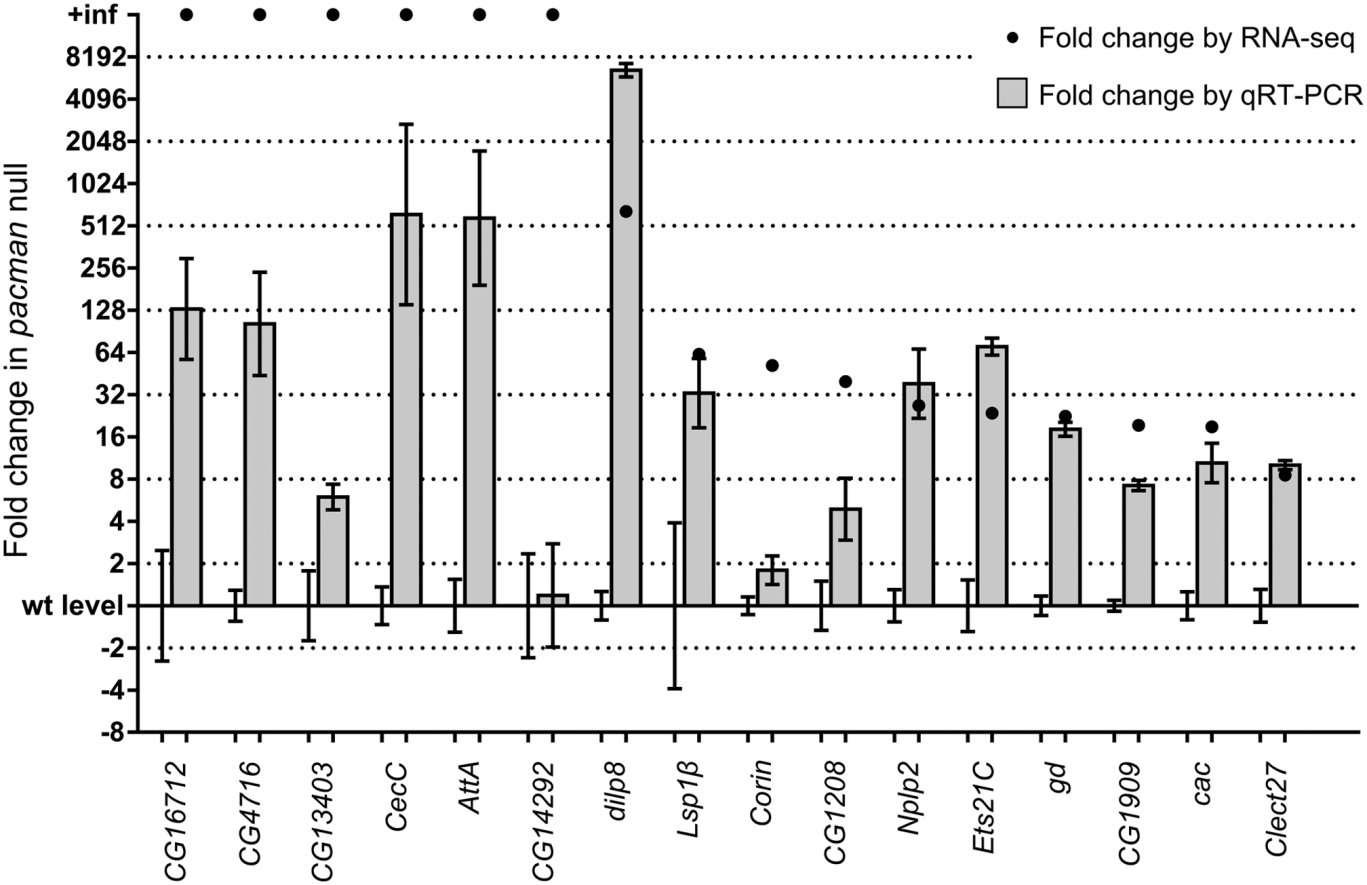
qRT-PCR verification of RNA-seq results. The expression differences of most of the 16 genes tested are similar by qRT-PCR and RNA-seq, although no genes were found to be infinitely upregulated by qRT-PCR. Only three genes were not significantly differentially regulated by qRT-PCR (*CG14292*, *Lsp1β* and *Corin*). The number of biological replicates and *P*-values for comparisons between wild-type level and *pacman* null level for each gene are shown in Table 3. Error bars show SEM.

Verification by qRT-PCR was performed on 16 shortlisted upregulated genes using RNA prepared from new *pcm^WT1^* and *pcm^14^* wing imaginal disc replicates and reverse transcribed using oligo-dT primers (Figure 6 and summarized in Table 3). Of the six genes reported as infinitely upregulated by Cuffdiff, *CG16712*, *CG4716*, *CecC* and *AttA* were also highly upregulated by qRT-PCR (>64-fold) while *CG13403* was only upregulated by 6.0-fold. *CG14292*, the least consistently expressed of the infinitely upregulated genes (*I* = 0.71), did not appear to be upregulated by qRT-PCR. The agreement between fold-change values for RNA-seq and qRT-PCR was relatively strong for mRNAs upregulated <64-fold with the fold-change values for *Nplp2*, *Ets21C*, *gd*, *CG1909*, *cac* and *Clect27* showing the strongest agreement between the RNA-seq and qRT-PCR techniques. *dilp8* showed the greatest upregulation by qRT-PCR (calculated as 6600-fold) and by RNA-seq (650-fold). The value for *dilp8* derived by qRT-PCR should be treated as an approximation, due to its unusual magnitude, but the upregulation of this gene in *pacman* null wing discs is clearly considerable. The levels of upregulation of *Lsp1β* were similar between RNA-seq and qRT-PCR, despite considerable inconsistency between replicates of each group (a *t*-test on the qRT-PCR data gives a *P*-value of 0.056). Of the remaining two genes, *CG1208* showed significant upregulation by qRT-PCR, but not to the same extent as by RNA-seq. *Corin* proved difficult to quantify accurately by qRT-PCR due either to its extremely low level of expression or to inefficiency of the TaqMan assay used to detect it. Notably, the RNA-seq data was consistent with our previous publication showing that the pro-apoptotic mRNA *reaper* is upregulated by 4.3-fold (Table 3). Therefore our qRT-PCR data is generally consistent with the RNA-seq data, particularly for genes which are upregulated less than 64-fold. The correlation between RNA-seq fold changes and qRT-PCR fold changes is analysed in Supplemental Figure S5.

**Table 3.** Summary of RNA-seq and qRT-PCR results for the 16 genes selected for verification

Gene	RNA-seq FC	Inconsistency index (I)	qRT-PCR FC	n (*pcm^WT1^*,*pcm^14^*)	*P*-value (*t*-test)	pre-mRNA FC	n (*pcm^WT1^*,*pcm^14^*)	*P*-value (*t*-test)
*CG16712*	+inf	0.61	130.8	6,6	0.0027			
*CG4716*	+inf	0.55	102.2	6,6	0.0004			
*CecC*	+inf	0.36	616.5	6,5	0.0012	103.1	5,5	0.0093
*CG13403*	+inf	0.26	6.0	6,6	0.0156			
*AttA*	+inf	0.68	580.6	6,6	0.0003			
*CG14292*	+inf	0.71	1.2	6,6	0.8936			
*dilp8*	650.1	0.49	6600.0	6,5	<0.0001	10.3	5,5	<0.0001
*Lsp1β*	62.1	0.80	32.8	6,5	0.056			
*Corin*	51.8	0.40	1.4	6,5	0.3128	61.7	5,5	0.0001
*CG1208*	39.7	0.58	4.9	6,6	0.0282			
*Nplp2*	26.8	0.47	38.2	6,6	0.0002	1.7	5,5	0.4925
*Ets21C*	23.6	0.40	70.3	6,6	<0.0001	29.5	5,5	<0.0001
*gd*	22.5	0.36	18.1	6,5	<0.0001			
*CG1909*	19.3	0.38	7.2	6,6	<0.0001			
*cac*	18.9	0.38	10.4	6,5	<0.0001			
*Clect27*	8.5	0.12	10.0	6,6	0.0002			
*rpr*	4.3	0.24	7.6*	6,6	<0.0001	1.7*	6,6	0.081

*indicates data from (15). *P*-values presented are from individual *t*-tests comparing *pacman* null transcript levels to wild-type and have not been adjusted for multiple comparisons.

### *Nplp2* and *dilp8* are post-transcriptionally upregulated in *pacman* null wing imaginal discs

**Figure 7.**
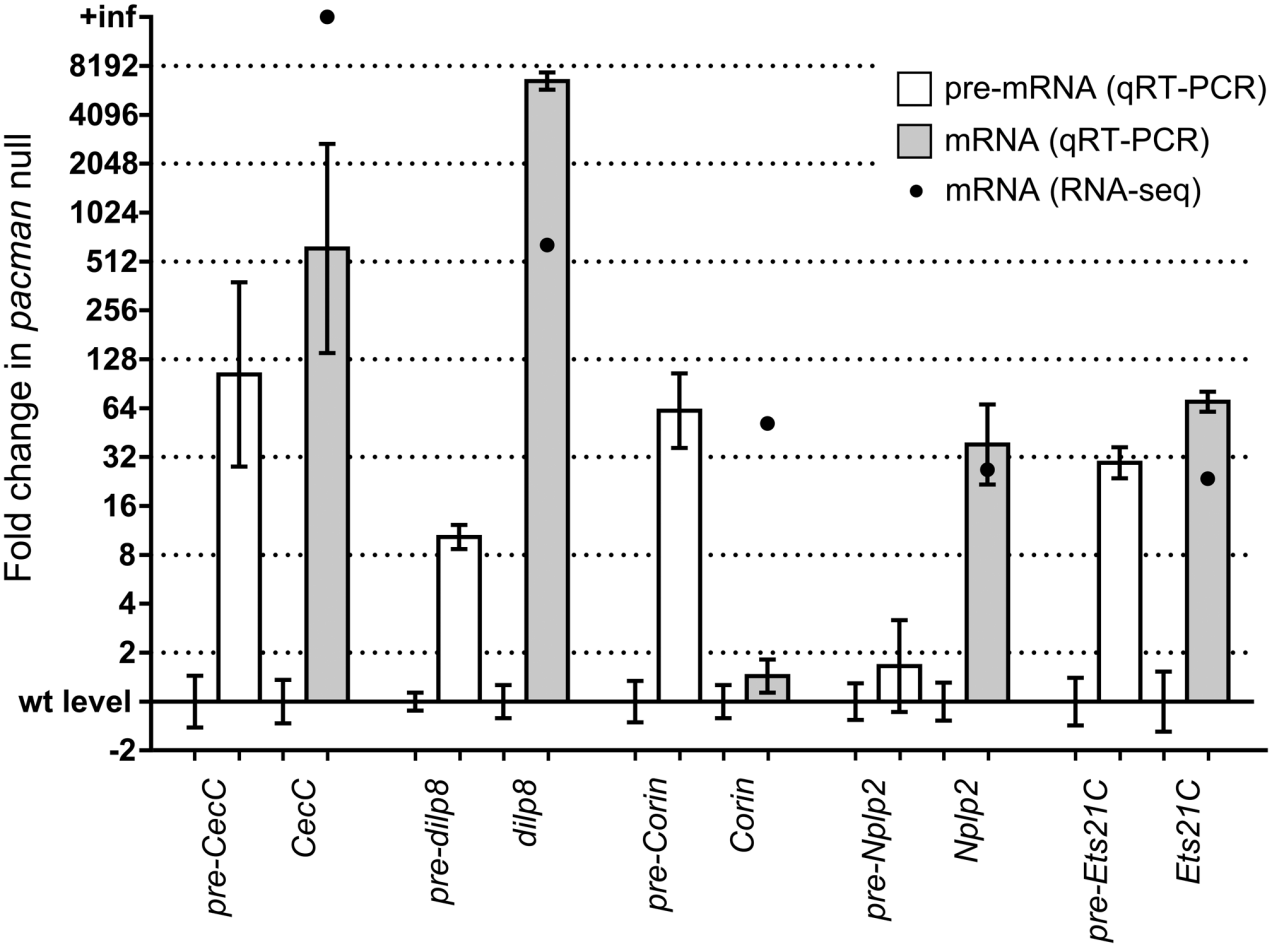
Determination of the level of regulation of *CecC*, *dilp8*, *Corin*, *Nplp2* and *Ets21C*. pre-mRNA levels were measured to determine if changes in expression were at the transcriptional level or at the post-transcriptional level. *pre-CecC* and *pre-Ets21C* increased to similar extents as their corresponding mRNAs, indicating transcriptional regulation of these genes. *pre-Corin* also increased to a similar level as *Corin* mRNA measured by RNA-seq. *pre-Nplp2* did not increase, indicating that regulation of *Nplp2* is post-transcriptional. *pre-dilp8* increased by a relatively small amount (10.3-fold) compared to the increase in *dilp8* mRNA (6600-fold by qRT-PCR or 650.1-fold by RNA-seq), indicating that regulation of *dilp8* is overwhelmingly post-transcriptional. The number of biological replicates and *P*-values for comparisons between wild-type level and *pacman* null level for each pre-mRNA/mRNA are shown in Table 3. Error bars show SEM.

The exoribonuclease Pacman is located in the cytoplasm, and acts on mRNAs rather than pre-mRNAs (3,4). The above genes that are upregulated at the mRNA level in *pacman* null mutants could be direct or indirect targets of Pacman. If they are direct targets, we would expect them to be upregulated at the mRNA level and not at the pre-mRNA level. Those genes upregulated at the pre-mRNA level as well as the mRNA level would be expected to be indirect targets, for example due to Pacman regulating an RNA encoding a transcriptional repressor. To determine possible direct targets of Pacman, we used the inconsistency index above to select five upregulated genes (*CecC*, *dilp8*, *Corin*, *Nplp2* and *Ets21C*) for further study. Custom TaqMan assays were designed to target their pre-mRNAs (Supplementary Figure S2) to determine whether their upregulation was due to transcriptional or post-transcriptional changes. cDNA was prepared from the *pcm^WT1^* and *pcm^14^* replicates used above with random primers. *CecC* and *Ets21C* both clearly showed regulation at the transcriptional level, as *pre-CecC* and *pre-Ets21C* increased in expression by similar amounts as the mature *CecC* and *Ets21C* mRNAs (Figure 7). *Corin* also showed an increase at the transcriptional level, as *pre-Corin* was increased in the *pacman* null wing discs, despite the level of mature *Corin* not increasing significantly by qRT-PCR (though the increase in *pre-Corin* by qRT-PCR was very similar to the increase in mature *Corin* by RNA-seq). *Nplp2* showed clear regulation at the post-transcriptional level, as the level of *pre-Nplp2* did not increase in the *pacman* null wing discs. *pre-dilp8* showed a relatively small but significant increase of 10.3-fold, suggesting partial regulation at the transcriptional level, however, when compared to the ∼6600-fold increase in mature *dilp8* mRNA, the regulation of *dilp8* appears to be overwhelmingly post-transcriptional. Therefore, of the four mRNAs tested, two (*dilp8* and *Nplp2*) are upregulated at the post-transcriptional level, indicating that they are targets of Pacman.

### Dilp8 is expressed in part of the wing pouch in *pacman* null wing imaginal discs

Although *dilp8* and *Nplp2* mRNAs increase substantially in expression in *pacman* null mutants compared to controls, this would only have biological significance if the mRNAs are functional and express protein. To test this we compared the levels of Dilp8 protein in *pacman* null and control wing imaginal discs using immunocytochemistry. An antibody to Nplp2 is not yet available. Our previous experiments (15) have shown that *pacman* null larvae are delayed in development (as L3 larvae) by around 32 hours compared to wild-type, so larvae used here were selected based on clearance of food from the gut, which occurs immediately before pupariation. Our previous results have also shown that *pacman* null wing imaginal discs undergo extensive apoptosis in the wing pouch (as marked by activated Caspase-3 staining) at this stage of development while wild-type wing imaginal discs do not (15).

**Figure 8.**
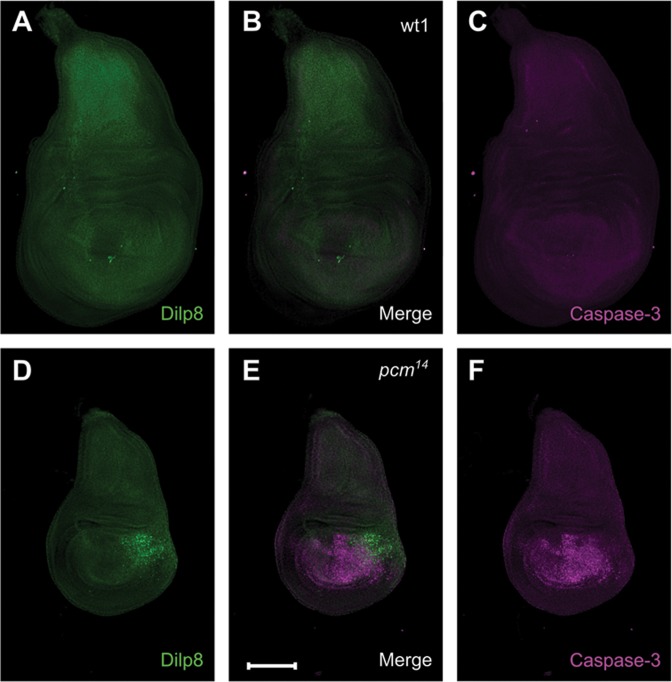
Immunocytochemistry for Dilp8 and activated Caspase-3 in *pcm^WT1^* and *pcm^14^* wing imaginal discs. (**A**–**C**) Apoptosis (magenta, visualized by the presence of activated Caspase 3) is not occurring in wild-type L3 wing imaginal discs immediately prior to pupariation and Dilp8 (green) is not expressed. (**D**–**F**) In *pcm^14^* wing discs at the same stage, apoptosis is apparent in the wing pouch area of the discs and Dilp8 is strongly expressed from a nearby region. Scale bar represents 100 μm.

**Figure 9.**
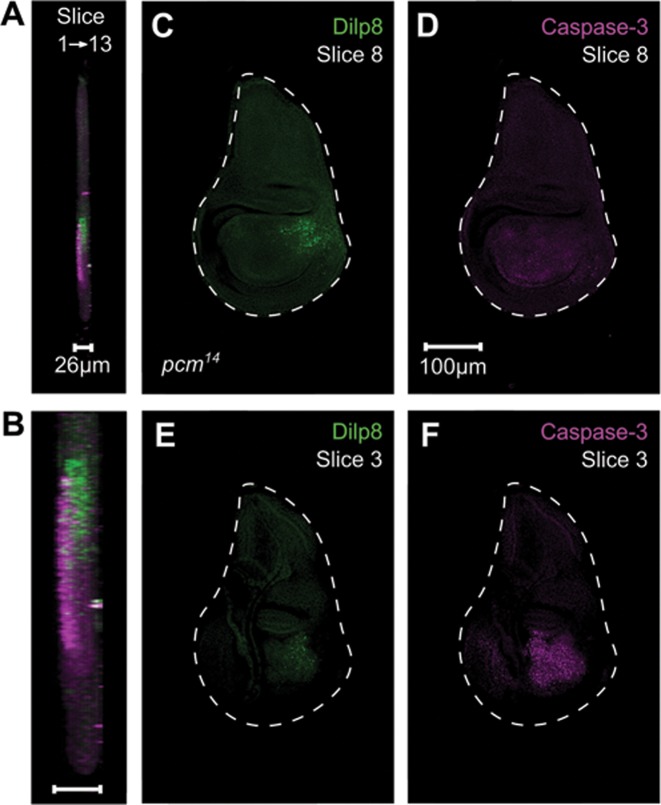
Activated Caspase-3 and Dilp8 are present at different levels in the *pacman* null wing imaginal discs. (**A**) Side view of a *pcm^14^* wing imaginal discs showing Dilp8 (green) and activated Caspase-3 (magenta). (**B**) Enlarged wing pouch of the wing imaginal disc. (**C** and **D**) Dilp8 is most strongly expressed around slice 8 of the wing disc (around 16 μm deep), where there is no activated Caspase-3 present. (**E** and **F**) Activated Caspase-3 is most strongly detected in slice 3 (6 μm deep) where Dilp8 expression is also detectable at a low level.

Dilp8 expression is undetectable above background level in wild-type L3 wing imaginal discs immediately prior to pupariation (Figure 8A). In *pacman* null (*pcm^14^*) wing imaginal discs at the same stage, Dilp8 expression is evident in part of the wing pouch at a much higher level than wild-type (Figure 8D). Therefore the increase in the level of *dilp8* mRNA is associated with an increase in Dilp8 protein expression. Activated Caspase-3 is also undetectable above background level in wild-type wing discs (Figure 8C) but is strongly detected in the wing pouch of *pcm^14^* wing discs (Figure 8F). Our data show that Dilp8 and activated Caspase-3 do not completely co-localize (Figure 8E). Viewed in 3D, Dilp8 expression is predominantly present at a different depth in the disc to activated Caspase-3 (Figure 9). This is consistent with the known function of Dilp8 as a secreted peptide which coordinates tissue growth with developmental timing (22,28).

### SUnSET labelling shows that the overall levels of protein synthesis are similar in *pcm^14^* wing imaginal discs compared to controls

**Figure 10.**
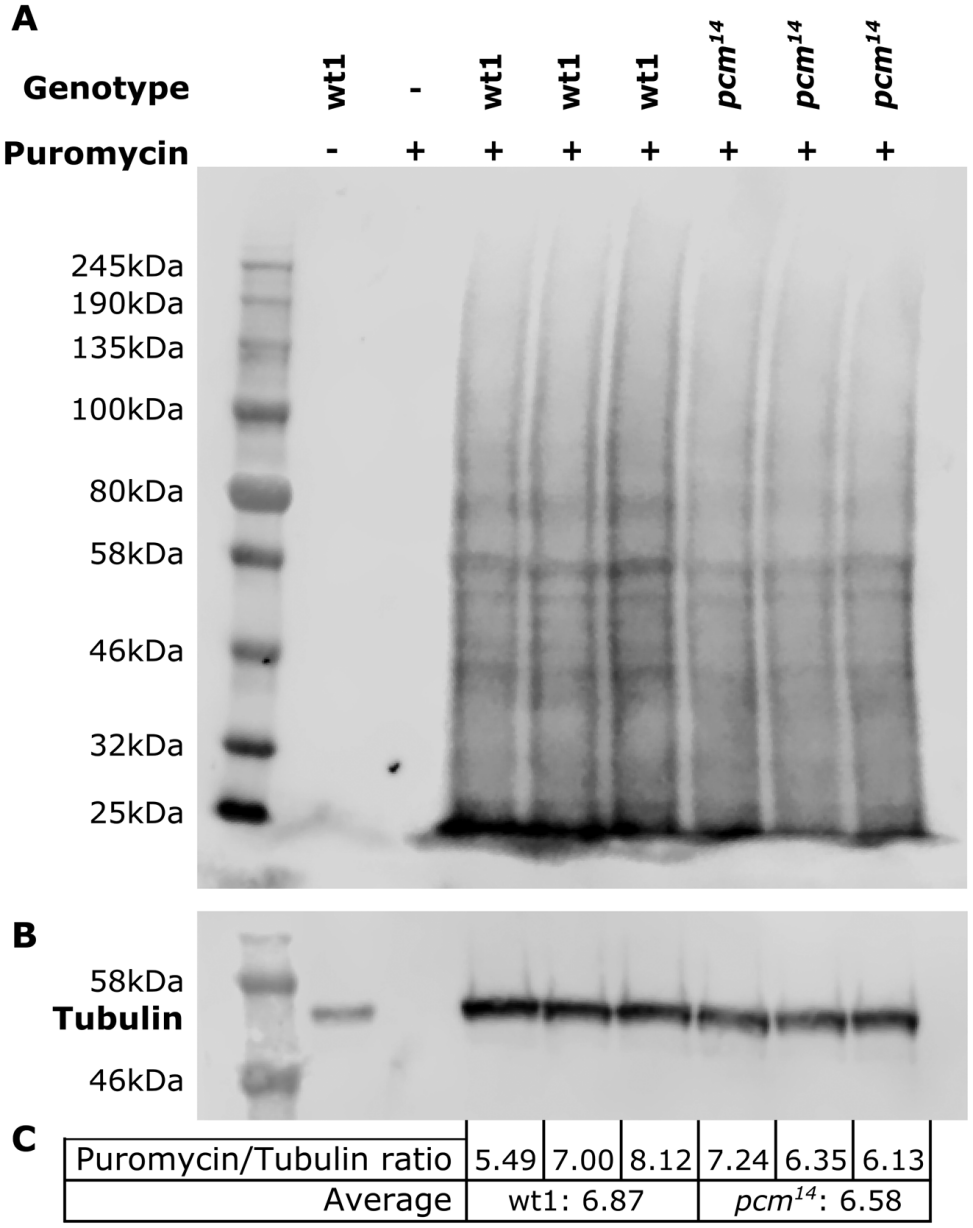
Quantification of global translation rates using SUnSET labelling. (**A-C**) Puromycin containing peptides are produced at the same rate for *pcm^WT1^* and *pcm^14^* when compared to Tubulin expression, indicating that increased *dilp8* expression in *pcm^14^* wing imaginal discs does not lead to a change in global translation rates.

Recent work on Dilp8 has shown that it is part of a gene regulatory network coordinating abnormal growth of a tissue to the overall growth programme. An increase in levels of *dilp8* in imaginal discs has previously been shown to be correlated with increased expression of *thor* mRNA (28). Thor (4E-BP) is known to bind to the cap-binding protein 4E and inhibit the initiation of protein translation (29) which is consistent with slower imaginal disc growth. Our RNA-seq data shows that *thor* transcript levels increase 5.2-fold in *pacman* null mutants compared to wild-type (Supplementary Figure S6). However, mammalian Insulin and Insulin-like growth factors have been shown to stimulate protein synthesis (30,31), so to determine whether global protein synthesis rates are affected in the *pcm^14^* mutant we used SUnSET labelling, where puromycin is incorporated into newly translated peptides and detected using a monoclonal antibody to puromycin (32). Although this technique has not previously been used in *Drosophila*, we have successfully carried it out on dissected *pcm^14^* and wild-type imaginal discs to show that protein synthesis does not vary overall (Figure 10). Puromycin was seen to be incorporated into peptides of all sizes with no difference in the global translation levels between *pcm^14^* and wild-type discs, indicating that the increase of Dilp8 is not stimulating or inhibiting overall protein synthesis rates. Detection of puromycin is specific to that incorporated into newly translated peptides as puromycin alone (Figure 10, lane 2) and wild-type wing discs in the absence of puromycin (lane 1) show no signal. Therefore the increased expression of Dilp8 does not lead to a global increase or decrease in translation, although Dilp8 could affect translation of specific transcripts.

## DISCUSSION

In this study we used RNA-seq in a global approach to identify candidate targets of the exoribonuclease Pacman during imaginal disc development. Using two *pacman* null mutants, together with their respective wild-type controls, we identified two new potential targets of Pacman; *dilp8* and *Nplp2*. These are upregulated 6600-fold and 38-fold respectively at the post-transcriptional level, as assessed by qRT-PCR. Dilp8 is a secreted insulin-like peptide which is known to co-ordinate tissue growth with developmental timing (22,28). The upregulation of Dilp8 protein, which we have visualized in L3 imaginal discs, is consistent with the developmental delay seen in the *pacman* null homozygous larvae. Nplp2 (neuropeptide-like precursor-2) transcripts are predicted to encode neuropeptides of 86 and 119 amino acids, the functions of which are unknown (18). Our analysis, together with our previous published results, suggests that transcripts encoding short peptides are particularly sensitive to regulation by Pacman. They also suggest that the role of this exoribonuclease is to suppress the expression of transcripts that are crucial in apoptosis and in endocrine signalling during normal development.

Our data highlights the importance of tightly controlled experiments using two null mutants, together with appropriate wild-type controls to improve the accuracy of the RNA-seq results and account for any differences in genetic background. Our RNA-seq results show that more genes are significantly upregulated >2-fold (488) than downregulated >2-fold (188) which is consistent with the exoribonuclease function of Pacman. We applied a novel ‘inconsistency index’ which allows the consistency of replicates to be expressed as a value between 0 and 1 without being biased by FPKM or expression changes between conditions, to narrow down the subset for validation by qRT-PCR. For those genes that are significantly upregulated >2-fold in the *pcm^14^* mutant by RNA-seq, the median locus size is 4306 bp (range 282–138 435 bp) whereas for genes significantly downregulated >2-fold, the median locus size is 6290 bp (range 214–202 218 bp). This suggests that Pacman tends to target shorter transcripts, though it should be noted that these results include both direct and indirect targets of Pacman and the proportion of each type is currently unknown. Although the GO enrichment scores are not high, they are consistent with the phenotypes observed in *pacman* mutants and suggest that Pacman affects a number of annotated cellular pathways in *Drosophila*.

Our data is consistent with upregulated RNAs being translated and functional as Dilp8 protein is expressed in *pcm^14^* wing imaginal discs (Figures 8 and 9). Therefore the *dilp8* transcripts upregulated in the *pcm^14^* mutants would appear to be capped and polyadenylated. This fits well with previous studies (33,34), where the family of Pacman/Xrn1 have been shown to include short linear motifs within their less structured C-terminal regions which bind co-factors involved in 5′-3′ degradation. In the case of *Drosophila* Pacman, the decapping activator Dcp1 binds a DCP1-binding motif within the C-terminal of the protein, thus linking decapping with 5′-3′ degradation. In a Pacman mutant, decapping would be expected to be severely inhibited resulting in upregulated transcripts which can be translated.

The results described in this paper are also in agreement with our previous publications using genetic approaches to analyse the effects of the *pcm^14^* mutant (15) and microarray analysis to identify the potential Pacman targets using *pcm^5^*, a hypomorphic allele (14). Supplementary Figure S6 shows that *reaper* is upregulated by 4.3-fold in the *pcm^14^* mutant by RNA-seq, which is in line with the 7.6-fold change in expression previously quantified by qRT-PCR (15). A comparison between our RNA-seq results and our previous microarray experiments performed on *pcm^5^* is shown in Supplementary Figure S7. These results show a similar upregulation for 10 mRNAs but no upregulation for *Dilp8* or *reaper*. However, relatively few genes changed significantly on the microarrays and the reason for the difference between these datasets is most likely to be both developmental and biochemical. Pacman is ∼66.6% functional in the *pcm^5^* mutant (15) and the wing imaginal discs are 81.7% the size of wild-type, allowing the pupae to develop into adults with smaller wings without a delay before pupariation. However, the imaginal discs in the *pcm^14^* null mutant are only 45.0% the size of wild-type, pupariation is significantly delayed and this mutation is completely lethal. It is therefore not surprising that the genes upregulated in the *pcm^5^* mutant represent only a subset of those upregulated in the *pcm^14^* mutant. It is highly likely that *dilp8, reaper* and other mRNAs are only upregulated once a Pacman function is reduced past a critical threshold. In addition, it is possible that the technical differences between microarray and RNA-seq could give rise to variability between these datasets.

Previous reports using the single cell organism *Saccharomyces cerevisiae* have shown that Xrn1 may buffer mRNA levels by entering the nucleus to interact with transcriptional repressors, thus reducing transcription (35,36). We have seen no evidence for this for Pacman in the multicellular organism *Drosophila*. In this and previous publications, we have used TaqMan assays designed to distinguish expression changes at the post-transcriptional level from changes at the transcriptional level (14–15,27). Therefore we can be confident that the RNAs we observe to be post-transcriptionally upregulated are not upregulated at the transcriptional level. Furthermore, we have never observed Pacman to be present in the nuclei of *Drosophila* cells (17). In any case, our experiments aimed to identify the transcripts whose overall expression is substantially perturbed in the *pcm^14^* mutant as these transcripts are more likely to result in the biological phenotypes observed. If there is a mechanism to maintain homeostasis for some transcripts then these transcripts are unlikely to cause the mutant phenotypes.

The finding that *dilp8* transcripts are highly upregulated in *pacman* null mutants is concordant with our previous results showing that these *pacman* mutations lead to a developmental delay in pupariation (15). The *Drosophila* insulin-like peptide family comprise eight members (Dilp1–Dilp8) which are secreted peptides required in development, growth, metabolism, stress responses and lifespan (37–39). These peptides display structural similarities to mammalian insulins, insulin-like growth factors and relaxins, including well-conserved positions of cysteines and disulfide bridges (40). One of the most studied peptides is Dilp2, which is produced by insulin producing cells in the brain of *Drosophila* and regulates developmental timing, body weight, lifespan, fecundity and trehalose levels, whereas Dilp6 plays a role in reallocating energy stores during pupation (41,42). Dilp8 has previously been reported to be highly induced in response to cellular damage (e.g. by gamma-ray irradiation) and in conditions where growth impairment produces a developmental delay (22,28). In regenerating tissues, Dilp8 is known to be secreted in vesicle-like structures from the imaginal discs (22) and remotely acts on the brain complex to suppress ecdysone production and activity and therefore delay the onset of pupariation (28). Previous publications have shown that tissue specific overexpression of Dilp8 results in developmental delay. For example, overexpression of *dilp8* using the *tubulin* promoter results in a pupariation delay of 55.9 h (28), whereas ectopic expression of *dilp8* using the disc specific *rotund* promoter delays pupariation by 2–3 days. These results are in line with the 32 h delay seen in *pcm^14^* mutants (15).

Our results suggest that the normal function of Pacman in L3 wing imaginal discs is to repress the expression of *dilp8*, so that the growth status of this tissue is coordinated with developmental timing. The effect of Pacman on *dilp8* transcripts in *Drosophila* is specific as *dilp6*, the only other member of the Dilp family expressed in wing imaginal discs, is unchanged at the RNA level in the mutant discs compared to the wild-type (Supplementary Figure S6). Since Pacman (XRN1) is highly conserved in humans and insulin-like peptides are also involved in tissue growth and homeostasis, it is possible that XRN1 regulates similar processes in humans.

**Figure 11.**
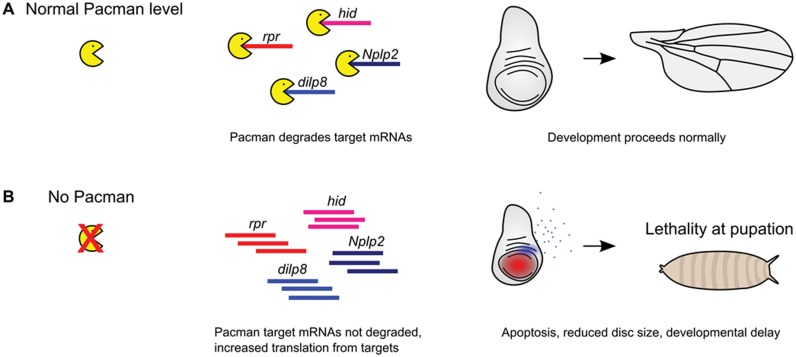
Model of Pacman function. (**A**) Under normal circumstances, Pacman degrades its target mRNAs to regulate their expression post-transcriptionally, which allows development to proceed normally. (**B**) Without Pacman, mRNAs of its targets are not degraded, allowing translation of apoptotic proteins such as Reaper and Hid, and signalling proteins such as Dilp8 and Nplp2. This results in a reduction of the size of imaginal discs, a delay in development and eventual lethality during pupation.

Previous work on Dilp8 has shown that it is part of a gene regulatory network coordinating abnormal growth of a tissue to the overall growth programme. An increase in levels of *dilp8* in imaginal discs has previously been shown to be correlated with increased expression of *thor* mRNA (28). Thor (4E-BP) is known to bind to the cap-binding protein 4E and inhibit the initiation of protein translation (29) which is consistent with slower imaginal disc growth. However, our SUnSET labelling experiments (Figure 10) show that global protein synthesis is unchanged in *pcm^14^* mutant wing imaginal discs compared to wild-type. However, we cannot rule out that there may be reductions in protein translation for specific mRNAs. Another mechanism involves induction of cell death by overexpression of the apoptotic gene *reaper* using *Beadex-Gal4*, which has been demonstrated to result in upregulation of *dilp8* transcripts by 13-fold in L3 wandering larvae (28). Our previously published results show that *pacman* null mutations result in a post-transcriptional upregulation of *reaper* by 7.8-fold at the RNA level and an increase in apoptosis in the wing pouch (15). The concomitant increase of *dilp8* transcript by 6600-fold is consistent with the idea that, in wild-type imaginal discs, Pacman preferentially degrades both *dilp8* and *reaper* transcripts to control both tissue growth and apoptosis (Figure 11).

The results detailed in this paper, together with our previous findings, show that Pacman regulates *dilp8*, *Nplp2* and *reaper* transcripts at the post-transcriptional level. These transcripts range from 452–901 nt in length suggesting that short transcripts encoding small peptides are particularly susceptible to degradation by Pacman. The mechanisms by which these transcripts are specifically regulated by Pacman or its homologues in natural tissues are not yet clear. However, work in tissue culture cells suggest that mRNAs may be targeted to Pacman/XRN1 by specific RNA binding proteins and/or miRNAs (3,43–44). In rat pancreatic cells, it is known that polypyrimidine tract-binding protein binds to a pyrimidine-rich region of the insulin 3′ UTR which contributes to the marked stability of insulin mRNA (45). Also, in rat cardiomyocytes, *miR-1* targets the 3′ UTR of IGF-1 reducing its expression (46). Therefore there are precedents for RNA-binding proteins/miRNAs regulating the expression of this family of proteins. Alternatively, mRNAs may be selectively sequestered in protein complexes which prevent access to ribonucleases and then be released for degradation via an unknown signal. The identification of a number of biologically relevant mRNAs regulated by Pacman presents an opportunity for understanding the mechanisms underlying the specificity of Pacman for certain mRNAs in a natural context.

## SUPPLEMENTARY DATA

Supplementary Data are available at NAR Online.

SUPPLEMENTARY DATA
