# Supplementary material for: RNA-seq reveals post-transcriptional regulation of Drosophila insulin-like peptide dilp8 and the neuropeptide-like precursor Nplp2 by the exoribonuclease Pacman/XRN1
Source: Nucleic Acids Res. 2015 Dec 9;44(1):267–80. doi: 10.1093/nar/gkv1336 (PMC4705666; doi:10.1093/nar/gkv1336)
Supplement: SUPPLEMENTARY DATA [file supp_44_1_267__index.html]

RNA-seq reveals post-transcriptional regulation of Drosophila insulin-like peptide dilp8 and the neuropeptide-like precursor Nplp2 by the exoribonuclease Pacman/XRN1 — SUPPLEMENTARY DATA 

# RNA-seq reveals post-transcriptional regulation of *Drosophila* insulin-like peptide *dilp8* and the neuropeptide-like precursor *Nplp2* by the exoribonuclease Pacman/XRN1

## SUPPLEMENTARY DATA

- SUPPLEMENTARY DATA
