# Supplementary material for: RNA-seq reveals post-transcriptional regulation of Drosophila insulin-like peptide dilp8 and the neuropeptide-like precursor Nplp2 by the exoribonuclease Pacman/XRN1
Source: Nucleic Acids Res. 2015 Dec 9;44(1):267–80. doi: 10.1093/nar/gkv1336 (PMC4705666; doi:10.1093/nar/gkv1336)
Supplement: SUPPLEMENTARY DATA [file supp_gkv1336_nar-02426-x-2015-File013.pdf]

Supplemental Figure 1

	Replicate	Paired reads (millions)	Unpaired reads (millions)	Total reads (millions)	Reads mapped
wild-type 1	1	14.12	0.44	14.57	88.6%
	2	12.55	0.32	12.87	87.0%
	3	14.06	0.43	14.49	84.8%
wild-type 2	1	11.67	0.36	12.04	85.3%
	2	14.70	0.60	15.31	84.6%
	3	15.17	0.48	15.65	84.5%
pcm^{14}	1	10.63	0.18	10.82	90.5%
	2	14.89	0.48	15.37	85.5%
	3	15.71	0.49	16.21	86.1%
pcm^{15}	1	14.71	0.37	15.08	85.1%
	2	14.90	0.33	15.24	85.3%
	3	16.14	0.32	16.47	86.2%

Summary of read counts and alignments for each RNA-seq replicate. Reads were aligned to chromosomes X, Y, 2, 3 and 4 of the FlyBase *Drosophila melanogaster* genome (r6.03) using TopHat v2.0.12 and Bowtie v2.2.3.

Program	Non-default parameters	Effect
TopHat2	-i 20	Minimum intron size 20bp (default 70bp).
	-I 150000	Maximum intron size 150,000bp (default 500,000bp).
	-r 100	Expected inner distance between mate pairs (default 50).
Cufflinks	-u	More accurate weighting of reads mapping to multiple locations.
	-N	Normalisation to upper quartile of number of fragments mapping to a locus instead of total number of fragments. Improves robustness of differential expression for lowly expressed transcripts.
	-compatible-hits-norm	Only fragments compatible with reference transcript counted in FPKM calculation.
Cuffmerge	None	
Cuffquant	-u	More accurate weighting of reads mapping to multiple locations.
Cuffdiff	-u	More accurate weighting of reads mapping to multiple locations.

Non-default parameters used for RNA-seq alignment and quantification.

Supplemental Figure 2

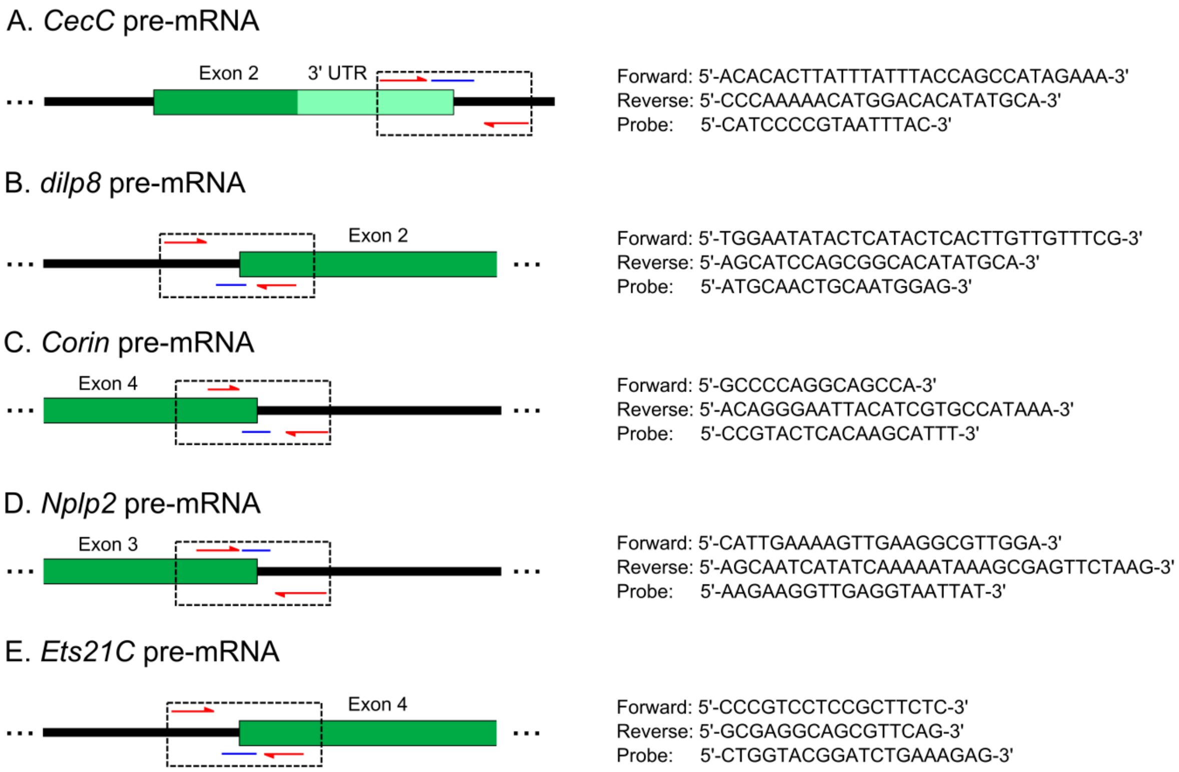

Custom TaqMan assays for pre-mRNA expression. Red lines indicate primers and blue lines indicate fluorescent probes.

Supplemental Figure 3

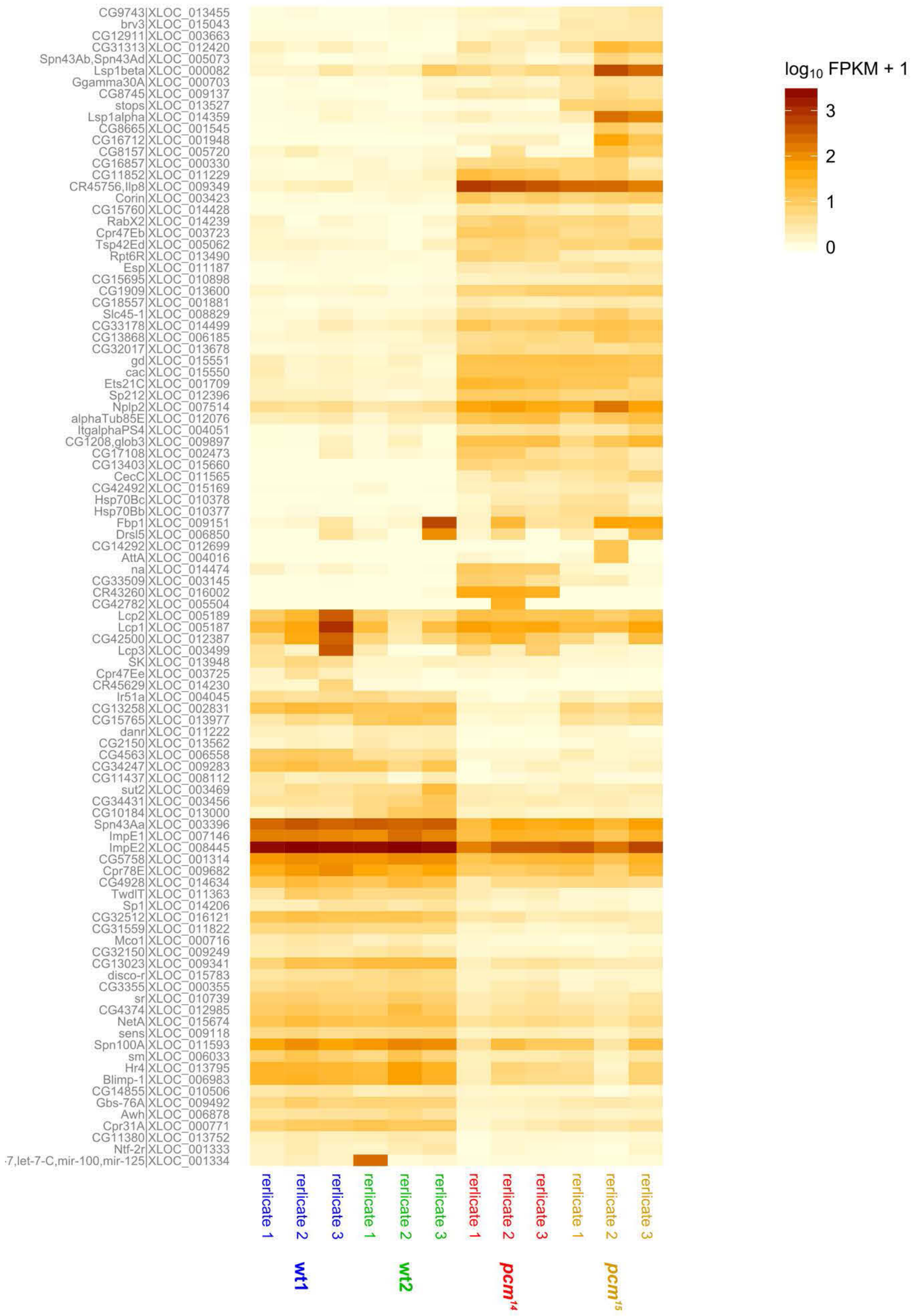

Heat map of top 50 up- and downregulated genes (produced using CummeRbund V2.0.0).

Supplemental Figure 4

Procedure for calculating the inconsistency index

For each gene,

$$I = \sqrt{\left(\frac{tSD_{WT}+tSD_{null}}{\sigma_{max}}\right)}$$

Where tSD_{WT} and tSD_{null} are the standard deviations of the transformed FPKM values for each group and σ_{max} is the largest value of tSD_{WT} + tSD_{null} in the experiment.

Firstly, FPKM values below 0.01 (including 0) are replaced with 0.01. The raw FPKM values are then transformed to have a mean of 1 by dividing each FPKM value by the group mean (tFPKM). The standard deviation of the transformed values is then calculated for each group (tSD). The tSDs for each group are added together (tSD_{WT} + tSD_{null}), with a lower value indicating greater gene expression consistency between replicates. To make the value more readily interpretable, it is scaled to be between 0 and 1 by dividing by the largest tSD_{WT} + tSD_{null} value in the experiment (that is, the value for the least consistently expressed gene in the experiment. In this case, σ_{max} = 4.89). Finally, a square root transformation is applied to even the spread of values between 0 and 1 while maintaining the same range.

This method allows quantification of the consistency of gene expression for individual genes without bias due to expression level or expression differences between conditions. It can easily be expanded to accommodate experiments with more than two groups.

Genes not expressed in any replicates will have 0 inconsistency as can easily be removed if required.

Worked example

	dilp8 wild-type FPKMs					
Raw	0.38	0.91	1.16	0.31	0.42	0.55
Raw mean	0.62					
$tFPKM_{WT}$	0.61	1.46	1.87	0.49	0.67	0.89
$tFPKM_{WT}$ mean	1.00					
tSD_{WT}	0.55					

	dilp8 pacman null FPKMs					
Raw	781.23	598.91	397.60	257.16	239.58	140.80
Raw mean	402.55					
$tFPKM_{null}$	1.94	1.49	0.99	0.64	0.60	0.35
$tFPKM_{null}$ mean	1.00					
tSD_{null}	0.61					

tSD_{WT} + tSD_{null} = 1.16

1.16/ σ_{max} = 1.16/4.89 = 0.23

dilp8 I = √0.2372 = 0.49

Distribution of I values

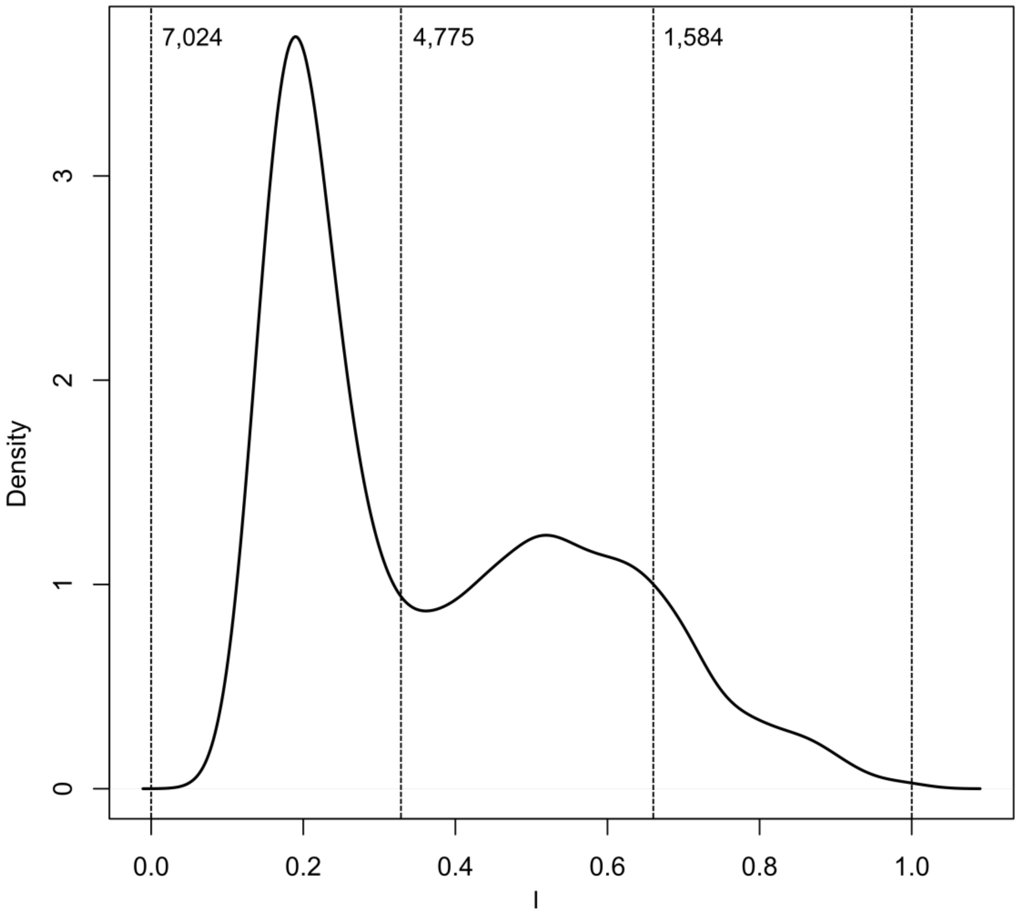

Of 13,383 genes detected in this study, 7,024 showed low inconsistency (0<I<0.33), 4,775 showed moderate inconsistency (0.33<I<0.66) and 1,584 showed high inconsistency (0.66<I<1).

Correlation between I and FPKM

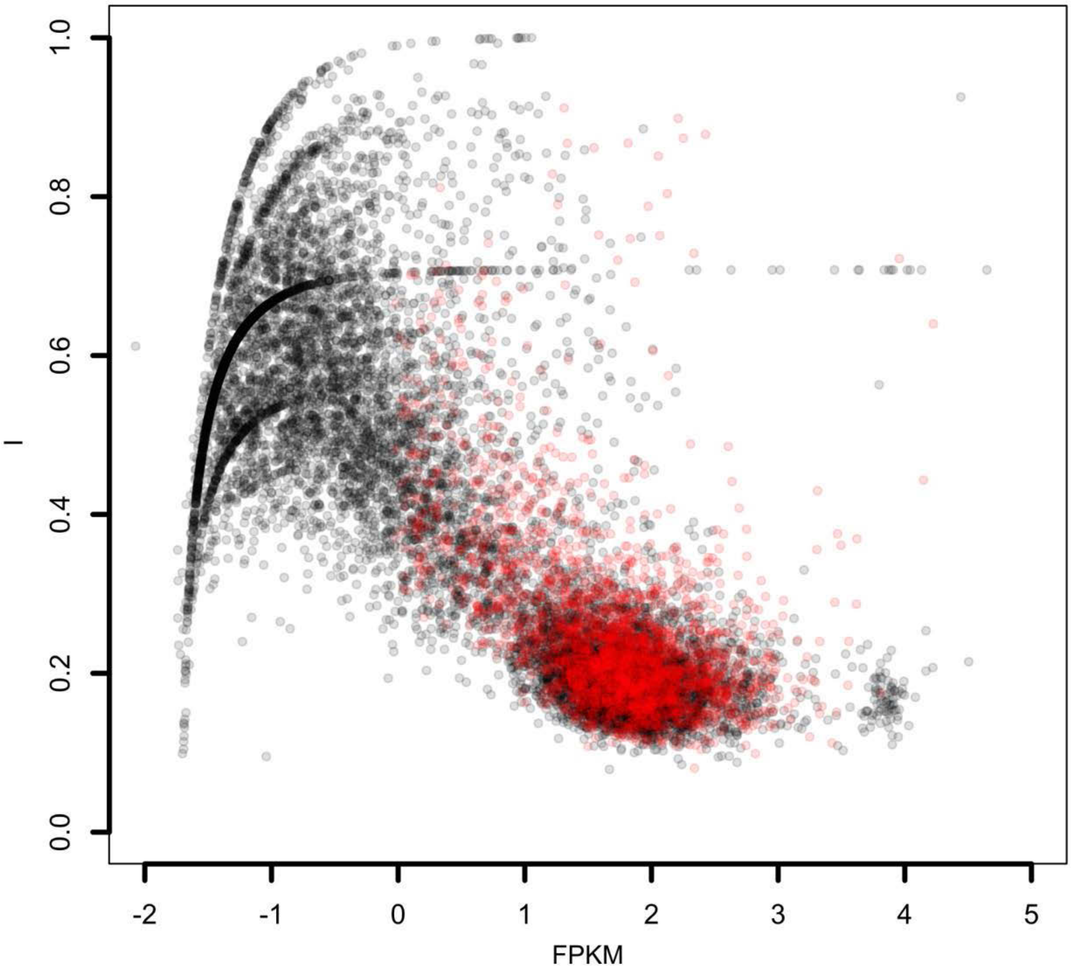

I correlates strongly with FPKM (r=-0.78). In general, more highly expressed genes (FPKM>1) are expressed more consistently than lowly expressed genes (FPKM <1). Significantly differentially expressed genes are marked in red.

Supplemental Figure 5

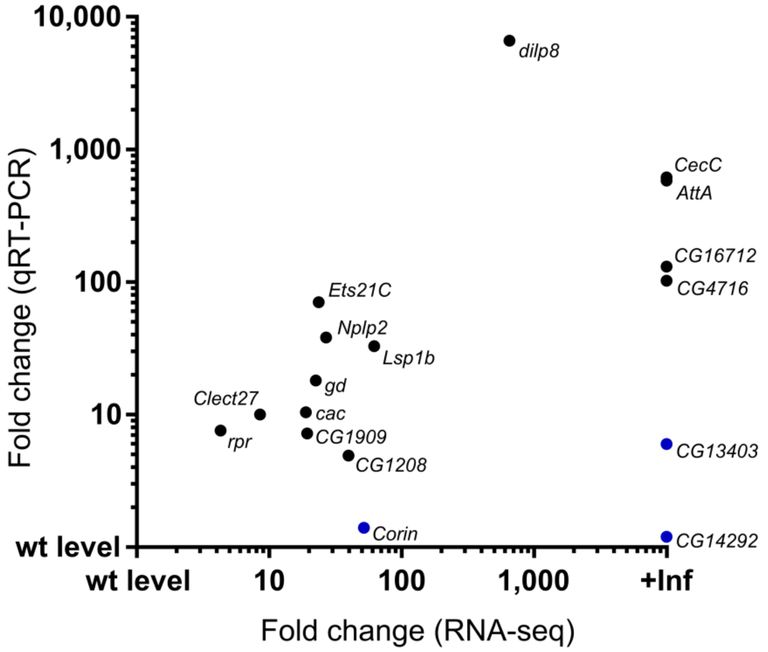

Scatter plot comparing fold changes in *pacman* nulls determined by RNA-seq and qRT-PCR. For most mRNAs (black points) correlation between the RNA-seq and qRT-PCR changes is strong (Spearman's r=0.77, p=0.0021). It was not possible to verify the expression levels of *Corin*, *CG13403* or *CG14292* (blue points) using qRT-PCR and if these are included, overall correlation between the two chemistries is lost (Spearman's r=-0.31, p=0.2249). This highlights the importance of confirming gene expression changes using more than one approach.

Supplemental Figure 6

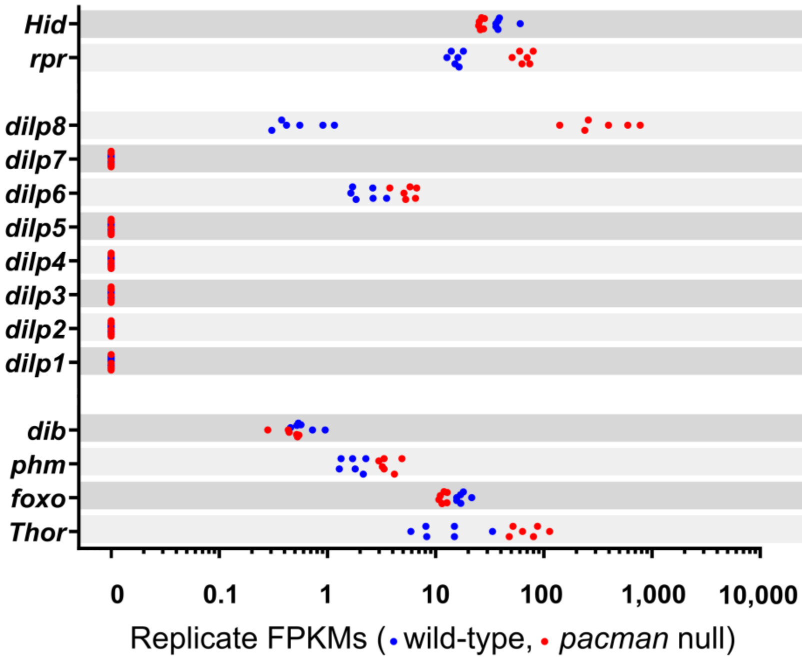

	I	Fold change	Significant?
Hid	0.24	-1.6	No
rpr	0.24	+4.3	Yes
dilp8	0.49	+650	Yes
dilp7	0	n.a.	n.a.
dilp6	0.32	+2.4	No
dilp5	0	n.a.	n.a.
dilp4	0	n.a.	n.a.
dilp3	0	n.a.	n.a.
dilp2	0	n.a.	n.a.
dilp1	0	n.a.	n.a.
dib	0.32	-1.4	No
phm	0.30	+2.1	Yes
foxo	0.20	-1.5	Yes
Thor	0.46	+5.2	Yes

Expression of genes linked to *Dilp8* in wild-type and *pacman* null wing imaginal discs by RNA-seq. "Significant" column indicates whether genes are reported as significantly differentially regulated by Cufflinks (corrected p-value <0.05).

Supplemental Figure 7

Concordant genes		
Gene	*pcm*^{5} fold change	*pcm* null fold change
CG5326	+3.31	+1.39
Hsp26	+2.83	+3.23
CG5953	+2.21	+5.54
CG7054	+2.13	+1.68
scramb1	+1.61	+1.41
CG32039	+1.56	+1.71
CG10407	+1.54	+1.53
CG10098	+1.49	+1.39
CG5044	+1.43	+1.80
kraken	+1.24	+1.34
trx	-1.34	-1.43
ea	-1.44	-1.43
CG32856	-1.57	-1.48
tutl	-1.73	-1.68
CG11263	-1.81	-2.62
CG13053	-2.12	-2.34
CG34247	-2.20	-20.39
Hrb87F	-2.28	-1.42

Discordant genes		
Gene	*pcm*^{5} fold change	*pcm* null fold change
simj	+1.87	-1.60
CG12054	+1.45	-1.22
CkIIalpha-i3	+1.82	-2.12
CG5873	+1.58	-2.36
CG13258	+1.52	-5.83
CG15282	+1.48	-1.36
CG6353	-1.54	+1.76
CG14314	-1.55	+2.26
CG6310	-1.66	+1.48
CG32364	-10.22	+2.75

Comparison of gene expression changes in *pcm*^{5} wing imaginal discs determined by microarray to gene expression changes in *pacman* null wing imaginal discs determined by RNA-seq.
